# Detecting response shift in health-related quality of life measurement among patients with hypertension using structural equation modeling

**DOI:** 10.1186/s12955-021-01732-w

**Published:** 2021-03-17

**Authors:** Hao Chen, Lin Zhu, Rui Zhou, Panpan Liu, Xiaoyang Lu, Donald L. Patrick, Todd C. Edwards, Hongmei Wang

**Affiliations:** 1 Department of Social Medicine of School of Public Health, Zhejiang University School of Medicine, 866 Yuhangtang Road, Hangzhou, 310058 Zhejiang People’s Republic of China; 2 Department of Public Health, Hangzhou Medical College, 481 Binwen Road, Hangzhou, 310051 Zhejiang People’s Republic of China; 3 Department of Pharmacy of the First Affiliated Hospital, Zhejiang University School of Medicine, 79 Qingchun Road, Hangzhou, 310003 Zhejiang People’s Republic of China; 4 Department of Health Services, University of Washington, H670 Health Sciences Building, Box 357660, Seattle, WA 98195-7660 USA

**Keywords:** Health-related quality of life, Response shift, Structural equation modeling, Hypertension, SF-36

## Abstract

**Background:**

Outcomes derived from longitudinal self-reported health-related quality of life measures can be confounded by response shift. This study was aimed to detect response shift among patients with hypertension attending a community-based disease management program.

**Methods:**

240 consecutive consulting or follow-up patients with diagnosed hypertension were recruited. The Short Form 36-item Health Survey was self-administered at 12 community health service stations at baseline and four weeks after attending the program. The 4-step structural equation modeling approach assessed response shift.

**Results:**

Data from 203 (84.6%) patients were eligible for analyses (mean age 65.9 ± 10.8 years, 46.3% female). The results showed uniform recalibration of social functioning (${\upchi}_{\mathrm{SBdiff}}^{2}$(1) = 22.98, P < 0.001), and non-uniform recalibration of role limitations due to physical problems (${\upchi}_{\mathrm{SBdiff}}^{2}$(1) = 8.84, P = 0.003), and bodily pain (${\upchi}_{\mathrm{SBdiff}}^{2}$(1) = 17.41, P < 0.001). The effects of response shift on social functioning were calculated as “small” (effect-size = 0.35), but changed the observed changes from improvement (effect-size = 0.25) to slight deterioration (effect-size = -0.10). After accounting for the response shift effect, the general physical health of participants was improved (effect-size = 0.37), while deterioration (effect-size = -0.21) in the general mental health was also found.

**Conclusions:**

Recalibration existed among patients with hypertension attending the disease management program. The interventions in the program might act as a catalyst that induced the response shift. We conclude that response shift should be considered in hypertension research with longitudinal health-related quality of life data.

**Supplementary Information:**

The online version contains supplementary material available at 10.1186/s12955-021-01732-w.

## Background

Health-related quality of life (HRQOL), representing people’s subjective assessment of their sense of health-specific well-being, has been frequently used as a health indicator in medical interventions or health surveys. However, the measurement of change in HRQOL may be affected by the fact that individuals’ frame of reference (or standard) or the concept and meaning of HRQOL can differ over time, known as Response Shift (RS) [1, 2]. A theoretical model (Additional file 1: Figure S1) of RS have been proposed by Sprangers and Schwartz, who postulated a dynamic feedback loop, where “catalyst,” “antecedents,” “mechanisms” and “RS” interacted and eventually maintained or led to changes in HRQOL [1], among which, RS was defined as three different types: (a) recalibration: a change in the respondent's internal standards of measurement; (b) reprioritization: a change in the respondent's values; and (c) reconceptualization: a redefinition of the target construct. With the existence of RS, individual experience of improvement or deterioration over time will be modified. In other words, substantial change of HRQOL can be over-or under-estimated without adjusting for RS [3–5]. Therefore, it is important to consider RS effect when measuring changes in HRQOL [6].

A variety of methodological methods are available to detect and adjust RS [2]. Based on the latent variable measurement modeling, Oort et al. [7, 8] have proposed a 4-step RS detecting procedure for longitudinal measurement occasions, named the Oort’s structural equation modeling (SEM) approach. The invariance of the corresponding parameters’ magnitude or model patterns across occasions was associated with the interpretation of all three RS types. Due to its versatility, Oort’s SEM approach has become the most widely used statistical method in RS detection [9].

Hypertension is a common chronic disease and a major risk factor for cardiovascular chronic diseases [10]. Many studies have confirmed that hypertension has been an influencing factor for the deterioration of HRQOL [11–14]. Previous research has provided evidence that recalibration type of RS existed among hypertensive male subjects [15] and hypertensive patients with coronary artery disease (CAD) [16]. In contrast, there is still a dearth of research that addresses RS phenomena against general hypertensive patients.

The community disease management program is a component of the national essential public health services in China, providing disease screening, drug therapy, long-term follow-up, and health education services to improve hypertension care [17]. Previous studies have found that patients with multiple chronic diseases changed perspective on their health status after attending self-management courses [18, 19]. The disease management program interventions may act as catalysts for psychological coping mechanisms, and subsequently affect how individuals think about their quality of life, which could in turn induce RS [20].

This study’s objective was to detect the effects of RS on HRQOL changes in patients with hypertension involved in the community disease management program. We hypothesized that patients who undergo this program would experience RS.

## Methods

### Study design and samples

The present study was a phase two study of a research project on quality of life of patients with common chronic diseases in the community health service and disease management strategy. In phase one of this project, the quality of life of consulting patients in the community health service was compared with those of the general population. Experimental studies of disease management for hypertension or diabetes patients were conducted in phase three [21–24].

The study cohort recruited 240 patients with hypertension in the community disease management program from a community health service center in Hangzhou, China. On the given week chosen by the study, all visiting or followed up patients in the belonged 12 community health service stations were invited to self-administer a health status survey before consultation until the quota for each station (n = 20) was met (baseline). Four weeks later, the participants were asked to complete the survey once again (follow-up). Patients were eligible if they (1) had a diagnosed hypertension (SBP ≥ 140 mmHg and/or DBP ≥ 90 mmHg), (2) were included in the community disease management program, and (3) had a regular follow-up. Patients with cognitive or visual problems or were unable to complete the questionnaires independently were excluded. The Ethics Committee of Zhejiang University School of Medicine approved the protocol, and all participants provided written informed consent.

### Measures

We used a validated Chinese (mainland) version of the Short-Form Health Survey (SF-36) to evaluate the HRQOL of patients with hypertension in this study [25]. The SF-36 instrument consists of 36 items which measure eight scales: physical functioning (PF), role limitations due to physical problems (RP), bodily pain (BP), general health (GH), vitality (VT), social functioning (SF), role limitations due to emotional problems (RE), and mental health (MH). All original scales were linearly transformed to a scale from 0 to 100, with a higher score indicating better HRQOL [26]. The demographic and disease information, including age, gender, marital status, employment status, educational attainment, health insurance, self-reported severe illness experience, duration of disease, blood pressure, health risk level (evaluated based on blood pressure, risk factors, target organ damage/diabetes mellitus, and multi-morbidities [17]), and medicine taken were also collected.

### Analyses

#### Structural equation modeling

The Oort’s SEM approach was applied to detect RS in a 4-step procedure [7, 8]: (1) establishing a decent measurement model; (2) forming a no RS model; (3) detecting RS; (4) evaluating adjusted change.

Step 1: in this step, we first established a 2-factor construct model for baseline data guided by exploratory factor analyses (EFA) and the published principal components analyses of the SF-36 [26]. We further applied it at both baseline (T1) and follow-up (T2), arrived at a longitudinal model. Combined with the guidance of modification index and substantive consideration, we modified it to develop the measurement model (Model 1). In model 1, all RS-related parameters (residual variances, intercepts, factor loadings) could be estimated freely across occasions. The common factors means and variances were constrained at zero and one respectively, as the scales and origins for the unobserved variables (i.e., common factors). Only when model 1 shows an acceptable fit could further analyses be conducted. A variety of model fit indexes were used to assess the appropriateness of model fit. These include the comparative fit index (CFI), the Tucker-Lewis index (TLI), the standardized root mean square residual (SRMR), and the root mean square error of approximation (RMSEA). With CFI, TLI>0.9, SRMR<0.1, and RMSEA<0.08 indicate acceptable model fit [27, 28].

Step 2: the invariance constraints on all RS-related parameters were placed across occasions (i.e., setting the paired parameters equal over time), forming the no RS model (Model 2). By using the ${\upchi}^{2}$ difference test, the overall existence of RS could be detected. If the model fit of model 2 was significantly worse than model 1, we could conclude that RS existed.

Step 3: in the third step, RS was detected by checking whether the fit of Model 2 can be improved significantly by releasing across occasion invariance constraints on RS-related parameters. Guided by the modification index, the across-occasion invariance of RS-related parameters was tested by the ${\upchi}^{2}$ difference test step-by-step. Invariance constraints that had been proved untenable (i.e., releasing these constraints led to significant improvement in model fitting) were removed individually, leading to a model (Model 3) where no modification index of RS-related parameters indicated a significantly better fitting (i.e., all RS was taken into account). Different types of RS were operationalized by the following parameters that varied across occasions: reconceptualization (factor patterns), reprioritization (factor loadings), uniform recalibration (intercepts), ununiform recalibration (residual variances). Given the backward approach could result in over identification of RS, a Bonferroni-adjusted critical value of 0.05/8 was used to control type I error [16, 29, 30].

Step 4: as the final step, all invariance constraints of common factors means, variances, and correlations were tested by checking if they can improve the fit of model 3. Tenable constraints were placed on the final model (Model 4). In this model, the adjusted change was assessed by testing the invariant hypothesis of common factor means across occasions after accounting for all RS. The estimated parameters in model 4 were used to calculate the effect-sizes of RS and the adjusted change. Effect size values of Cohen’s d = 0.2, 0.5, and 0.8 are considered “small”, “medium”, and “large” [31].

### Statistical analyses

Descriptive statistics were used to characterize the study sample. We then applied *t* test, one-way ANOVA, Chi-square, or Fisher’s Exact tests to compare demographic, health conditions, and HRQOL scores on a subsample with baseline data only or missing data (the attrition sample) and one with eligible data (the analytic sample) to examine selection bias. These analyses were implemented using IBM SPSS version 24.

Mplus version 7.4 was used for the SEM analyses. Given our data deviated from multivariate normality, the Robust Maximum Likelihood Estimator (MLR) was employed as the estimator [32]. When conducting the ${\upchi}^{2}$ difference tests in Mplus with the MLR estimator, it was essential to adjust the ${\upchi}^{2}$ statistics. The Satorra-Bentler scaling correction was conducted for ${\upchi}^{2}$ statistics adjustment [33].

## Results

### Participants’ characteristics and health information

The initial cohort included 240 patients. 211 (87.9%) patients completed the questionnaire twice, while 8 patients with missing data were excluded, resulting in a data set of 203 (84.6%) patients used for analyses. Among 203 patients with eligible data, 94 were female (46.3%), and the mean age was 65.9 years (range 35–86, SD 10.8). About one fifth (19.2%) of participants had college or above education. Four fifths (75.4%) of participants were unemployed or retired. More than four fifths (83.7%) of participants had an annual household income of no more than 60,000 RMB. Seven (3.3%) participants did not have any health insurance (Table 1).

**Table 1:** Demographic characteristics of study participants

Variable	N	n (%)
*Gender*	203	
Female		94 (46.3)
Male		109 (53.7)
*Marital status*	201	
Married/co-habiting		181 (89.2)
Other		20 (9.6)
*Education attainment*	201	
No school		6 (3.0)
Elementary school		55 (27.1)
Middle school		60 (29.6)
High school or vocational training		41 (20.2)
College or above		39 (19.2)
*Employment status*	191	
Employed		30 (14.8)
Not employed/retired		153 (75.4)
Full-time housework		8 (3.9)
*Annual household income ($1US* = *6.8Yuan)*	203	
< 60,000 RMB		170 (83.7)
≥ 60,000 RMB		27 (13.3)
Health insurance	203	
Urban employee basic medical insurance		80 (39.4)
Urban resident basic medical insurance		107 (52.7)
Other insurances ^a^		9 (4.3)
No insurance		7 (3.3)

^a^Other insurances include new cooperative medical scheme, commercial medical insurance, free medical service, etc.

About one of the third participants reported their own or family member’s serious disease experience. Sixteen (7.8%) participants had hypertension no more than six months. More than half (54.2%) of participants had average blood pressure lower than 140/90 mmHg. About three fourths (74.4%) of participants had a low or medium level of health risks. Sixteen (7.9%) participants did not take any medicine (Table 2).

**Table 2:** Health conditions of study participants

Variable	N	n (%)
Experienced severe illness	202	71 (35.0)
Family members experienced severe illness	201	68 (33.5)
*Duration of hypertension*	202	
< 6 months		16 (7.8)
≥ 6 months		186 (92.2)
*Last month blood pressure*	200	
< 140/90 mmHg		110 (54.2)
≥ 140/90 mmHg		90 (45.8)
*Health risk level* ^a^	186	
Low		51 (25.1)
Medium		100 (49.3)
High		26 (12.8)
Very high		9 (4.4)
*Anti-hypertensive medications*	202	
0		16 (7.9)
1		134 (66.0)
2		44 (21.7)
≥ 3		8 (4.0)

^a^Health risk was assessed based on blood pressure, risk factors, and target organ damage/diabetes mellitus, and multi-morbidities [17]

### Selection biases

The examination of differences between the attrition sample and analytic sample revealed that they were comparable on almost all demographic characteristics and health conditions. However, there were differences between the two samples, such that patients from the attrition sample were more likely to do full-time housework (P = 0.032). A comparison of HRQOL scores between the two samples revealed that the attrition sample reported slightly worse physical functioning (P = 0.028) and role limitations due to physical problems (P = 0.013) than the analytic sample (see Additional file 2: Table S1).

### Structural equation modeling

#### Measurement model

We fixed two factors in EFA according to the principal components model of the SF-36 scales described by Ware et al. [26]. The two common factors were named as GenPHYS (General physical health) and GenMENT (General mental health). The GenPHYS were measured by PF, RP, BP, RE, while the GenMENT were measured by GH, SF, VT, MH.

**Fig. 1:**
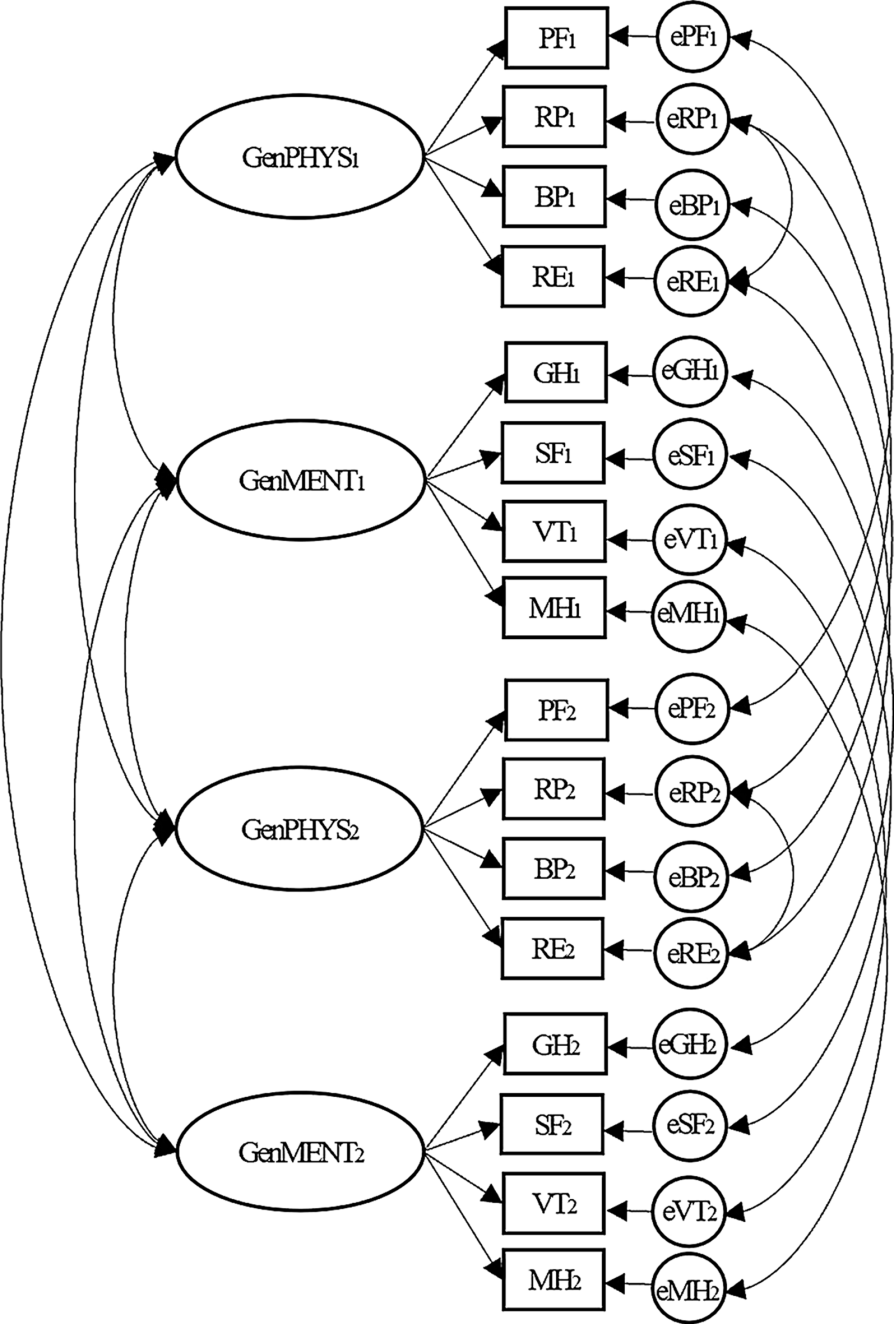
The measurement model used in RS detection

Figure 1 shows the measurement model resulted from EFA and substantial considerations. The ovals worded as GenPHYS and GenMENT represented latent variables. The rectangles represented the eight scales. The circles rightmost represented residual terms. The subscript numbers denoted measurement occasions (1 = baseline; 2 = follow-up). The straight arrows represented loadings, and the curved arrows represented covariances. The residual factors were correlated across occasions. The residual factors for RP and RE were correlated by the instruction of the modification index, as both scales have close meaning and wording expression about social roles.

#### Detection of RS and adjusted change

**Fig. 2:**
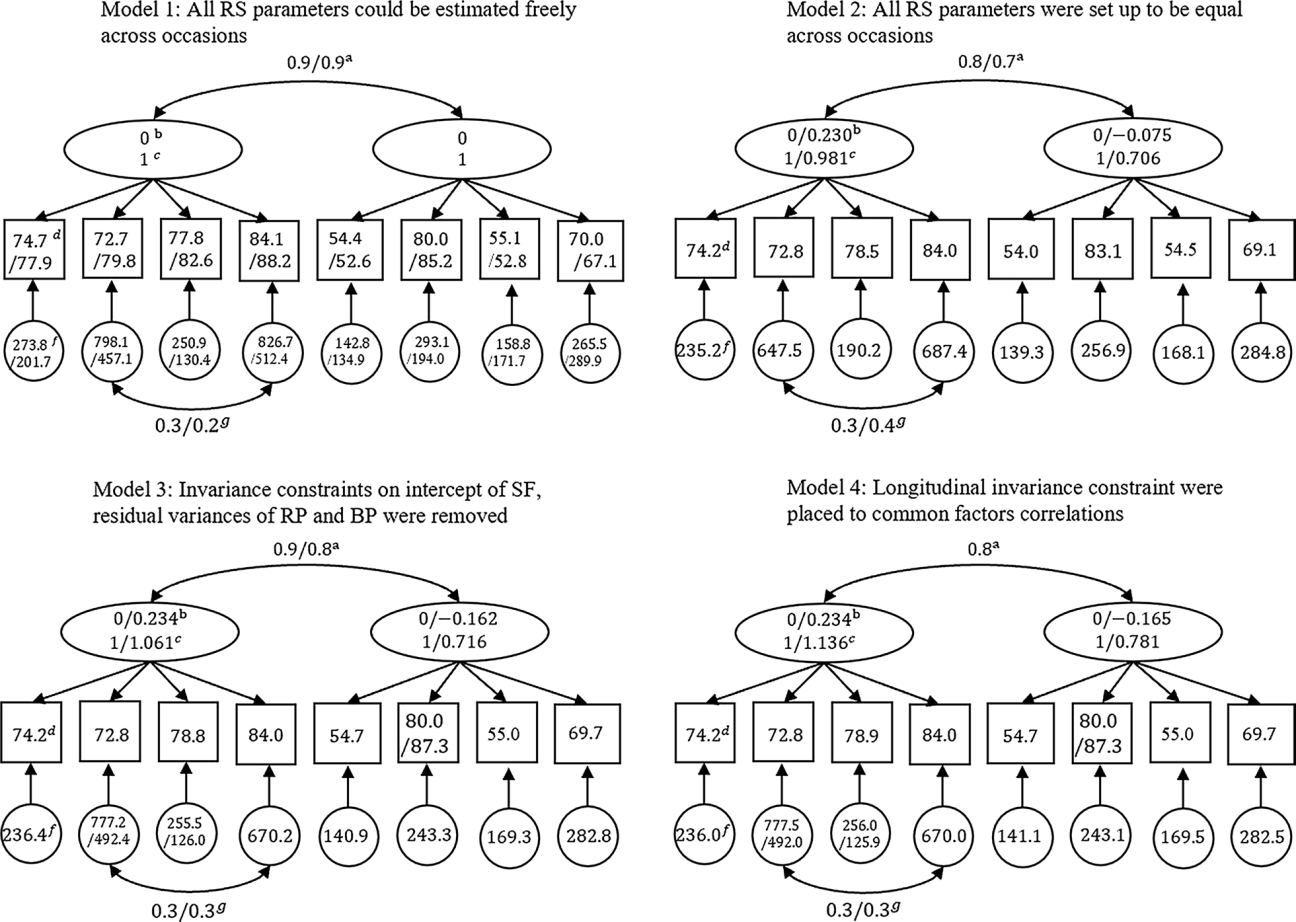
Simplified patterns of longitudinal models used in RS detection. Numbers are estimated parameters in each Model. They are ^a^common factor correlation, ^b^common factor means, ^c^common factor variances, ^d^intercepts, ^f^residual variances, and ^g^residual correlation. Common factor loadings are not revealed in these figures, since reprioritization and reconceptualization were not found in this sample. Parameters divided by a slash represent they can be estimated freely across occasions, and separate the first and second occasion estimates

Details for model fit are given in Table 3, and Fig. 2 provided simplified patterns of the 4 longitudinal models used in RS detection.

**Table 3:** Goodness of fit of models in the 4-step detection procedure

Model	Df	CHISQ	CFI	TLI	RMSEA (90%CI)	SRMR
Model 1	88	151.6	0.951	0.934	0.060 (0.043,0.075)	0.050
Model 2	108	222.6	0.912	0.903	0.072 (0.059,0.086)	0.067
Model 3	105	178.5	0.944	0.936	0.059 (0.044,0.073)	0.059
Model 4	106	179.7	0.944	0.936	0.059 (0.043,0.073)	0.060

Step 1: all fit indexes for model 1 were in an acceptable range, indicating an appropriate unconstrained measurement model was established.

Step 2: in model 2, all RS-related parameters were constrained to be invariant across occasions. The fit of model 2 was still acceptable but was significantly worse than model 1 (${\upchi}_{\mathrm{SBdiff}}^{2}$(20) = 69.53, P < 0.001), indicating the overall existence of RS.

Step 3: after controlling for Type I error (Bonferroni-adjusted critical value=0.006), constrains of the residual variances of RP (${\upchi}_{\mathrm{SBdiff}}^{2}$(1) = 8.84, P = 0.003) and BP (${\upchi}_{\mathrm{SBdiff}}^{2}$(1) = 17.41, P < 0.001), the intercept of SF (${\upchi}_{\mathrm{SBdiff}}^{2}$(1) = 22.98, P < 0.001) were removed, indicating non-uniform recalibration of RP and BP, uniform recalibration of SF.

Step 4: the invariance constrain on common factor correlations across occasions were placed on model 4. The longitudinal differences in both common factors means were significant, indicating a significant adjusted change in GenPHYS and GenMENT. The GenPHYS improved ( + 0.234, P < 0.001), whereas the GenMENT deteriorated (− 0.165, P = 0.025), with effect-sizes all considered “small” (effect-sizes = 0.37, − 0.21 respectively). Estimated parameters of model 4 are presented in Table 4.

**Table 4:** Parameters estimated in model 4

	Pre-test				Post-test			
	GenPHYS_1_		Gen-MENT_1_		GenPHYS_2_		Gen-MENT_2_	
*Factor loadings*								
PF	17.557				17.557			
RP	29.074				29.074			
BP	13.761				13.761			
RE	18.159				18.159			
GH			14.339				14.339	
SF			12.508				12.508	
VT			12.664				12.664	
MH			13.713				13.713	
Intercepts								

	PF	RP	BP	RE	GH	SF	VT	MH
Pre-test	74.214	72.804	78.853	83.962	54.656	80.049	55.038	69.664
Post-test	74.214	72.804	78.853	83.962	54.656	**87.290**	55.038	69.664
Residual variance								
	ResPF	ResRP	ResBP	ResRE	ResGH	ResSF	ResVT	ResMH
Pre-test	236.00	777.51	255.96	670.02	141.12	243.06	169.46	282.52
Post-test	236.00	**492.01**	**125.94**	670.02	141.12	243.06	169.46	282.52
Common factor variances								

	Pre-test				Post-test			
	GenPHYS_1_		Gen-MENT_1_		GenPHYS_2_		Gen-MENT_2_	
	1.00		1.00		**1.136**		**0.781**	
Common factor correlations								
Pre-test								
GenPHYS_1_	1							
Gen-MENT_1_	0.840		1					
Post-test								
GenPHYS_2_	0.871		0.685		1			
Gen-MENT_2_	0.561		0.583		0.840		1	
Common factor means								

	Pre-test				Post-test			
	GenPHYS		Gen-MENT		GenPHYS		Gen-MENT	
	0.00		0.00		**0.234 P < 0.001**		**-0.165 P = 0.025**	

Parameters of factor loadings are unstandardized; Results indicating across-measurement variance are printed in bold

Table 5 shows a significant test of RS, and the effect-sizes of observed change, RS, and adjusted change. RS in the SF, RP, and BP scale were all significant. The effect was calculated as “small” for uniform recalibration of SF (effect-size = 0.35). RP and BP’s effect-sizes were zero at the group level since the non-uniform recalibration indicated individual internal standard changes in different directions. After accounting for the RS effect, all scales except for the PF scale were stable. The PF scale of the participants improved slightly (effect-size = 0.21).

**Table 5:** Significance tests of RS, and the effect-sizes of observed change, RS, and adjusted change

Scale	RS	Significance test		Effect-sizes ^a^		
		${\upchi}_{\mathrm{SBdiff}}^{2}$(df = 1)	Prob	Observed change	RS	Adjusted change
PF				0.21		0.21
RP	Non-unif. recalibration	8.84	0.003	0.19	0.00	0.19
BP	Non-unif. recalibration	17.41	< 0.001	0.16	0.00	0.16
RE				0.12		0.13
GH				-0.13		-0.13
SF	Uniform recalibration	22.98	< 0.001	0.25	0.35	-0.10
VT				-0.12		-0.11
MH				-0.10		-0.10

^a^ Effect-sizes were calculated as corresponding parameters difference in model 4 divided by the estimated standard deviation;

Values of 0.2, 0.5, 0.8 indicate “small”, “medium”, and “large” effect-sizes. Values less than 0.2 are considered “negligible”;

Bonferroni-adjusted critical value = 0.006

## Discussion

This study explored the occurrence of RS in patients with hypertension attending the community disease management program by using Oort’s SEM approach. In our sample, we detected indications of recalibration in the SF, RP, and BP scale.

First, the intercept value of SF was higher at follow-up than baseline. This indicated that at follow-up, participants rated a better response category (higher scores) to social functioning than would be expected on account of the deterioration of their general mental health. The meaning of their response scale anchors of the SF scale may have been changed after the 4-weeks disease management program experiences, and this could be attributed to (uniformly) recalibration. Possible explanations for the result could be that the health education and social comparisons caused patients to alter their standards of comparison [20], and the reserve-building activities reduced health worries and increase calm and peaceful appraisal [34].

Second, the residual variances of RP and BP were lower at 4-weeks after baseline. This means the participants were less heterogeneous in what is specific to the measurement of role limitations due to physical problems and bodily pain. Therefore, some portion of changes in the observed variance of RP and BP cannot be explained by changes in the variability of the common factor (i.e., the general physical health), and it could be attributed to the (non-uniform) recalibration of a portion of the RP and BP response scale [8].

Since the non-uniform recalibration was defined as internal standard changes in different directions, the calculated effects on group level were absolutely zero. Whereas the effects of uniform recalibration in the SF scale were calculated as “small,” and the influence on the measurement results was noticeable, which emphasized the importance of consideration to RS in longitudinal HRQOL studies. After accounting for RS effects, we found a slight improvement in general physical health (GenPHYS) and a slight deterioration in general mental health (GenMENT).

Studies of RS in HRQOL research have grown steadily in more than two decades, while the arguments about what RS really is have never been stopped. Oort and colleagues [35] defined RS as a special case of bias both from measurement perspective and conceptual perspective, which must be accounted for to arrive at a “true change.” Whereas Rapkin and Schwartz treated RS as one kind of different changes in QOL, which were caused by the change of the way individual appraise his/her quality of life [36]. Nevertheless, they all agreed that RS effects should be treated as meaningful and distinct information rather than errors. We believe the RS effects should be recognized as an important and effective consequence derived from the disease management program and shall differ from the direct result that the interventions improved or deteriorated QOL, but rather mediated or moderated by the changes in the way individuals think about their QOL.

Prior research has explored the mechanisms where RS is produced. Sprangers and Schwartz [1] have proposed several mechanisms, including social comparisons and coping strategies. Empirical evidence has suggested that social comparison acted as a mediator between life events and RS [37]. Patients who considered themselves better off than others tend to maintain their quality of life even with a worsening functioning [38]. Oort inferred that learning how to cope with illness could induced RS in patients’ physical functioning [7]. By integrating the appraisal process into the theoretical model of RS, Rapkin and Schwartz proposed the concepts and methods for direct measurements of the individual psychological process involved in rating a QOL item [20], which provided a deep insight into how and when RS occurred.

The then-test method was used to detect RS for this cohort in our previous work [39]. The results deviated in the PF scale, where the then-test has found the recalibration. The reason could be that the then-test approach was susceptible to inaccurate recall [40], especially among elderly participants [41], while the SEM approach was immune to recall issues.

This study has some limitations that should be noted. Although the measurement model that we used fitted the data well with sufficient statistical power, we remain cautious that our analytic sample maybe not large enough to draw substantive conclusions about this specific population. Besides, our sample size maybe not large enough for further multiple group analyses to explore the possible predictors of response shift within the SEM framework. Second, the group level model-based methods identify RS in terms of the variances of model patterns or parameters, but RS may not the only reason that arises these differences. This explained why the direct measurement of individual coping mechanisms and appraisal process is necessary. As Rapkin and Schwartz [36] mentioned, linking these group-level statistical methods to appraisal is central to the (RS research) field, which will be explored in our future studies.

## Conclusions

In summary, this study identified the recalibration RS among patients with hypertension over a 4-week period of a disease management program. The RS effects on measurement results were significant and could be considered an important and complementary outcome derived from the intervention. Further studies with larger samples are necessary, and the direct measurement of the mechanism and appraisal process is recommended.

## Supplementary Information

**Additional file 1: Figure S1**. Theoretical model of RS.

**Additional file 2: Table S1**. The result of selection bias test at baseline.

## Data Availability

The datasets used and/or analyzed during the current study are available from the corresponding author on reasonable request.

## Abbreviations

RS: Response Shift
SEM: Structural Equation Modeling
HRQOL: Health-Related Quality of Life
CAD: Coronary Artery Disease
SF-36: 36-Item Short Form Health Survey
PF: Physical Functioning
RP: Role Limitations due to Physical Problems
BP: Bodily Pain
GH: General Health
VT: Vitality
SF: Social Functioning
RE: Role Limitations due to Emotional Problems
MH: Mental Health
SD: Standard Deviation
EFA: Exploratory Factor Analysis
MLR: Robust Maximum Likelihood Estimator
CFI: Comparative Fit Index
TLI: Tucker–Lewis Index
SRMR: Standardized Root Mean Square Residual
RMSEA: Root Mean Square Error of Approximation
GenPHYS: General Physical Health
GenMENT: General Mental Health
