# Supplementary figures and images for: Detecting response shift in health-related quality of life measurement among patients with hypertension using structural equation modeling
Source: Health Qual Life Outcomes. 2021 Mar 17;19:88. doi: 10.1186/s12955-021-01732-w (PMC7968327; doi:10.1186/s12955-021-01732-w)

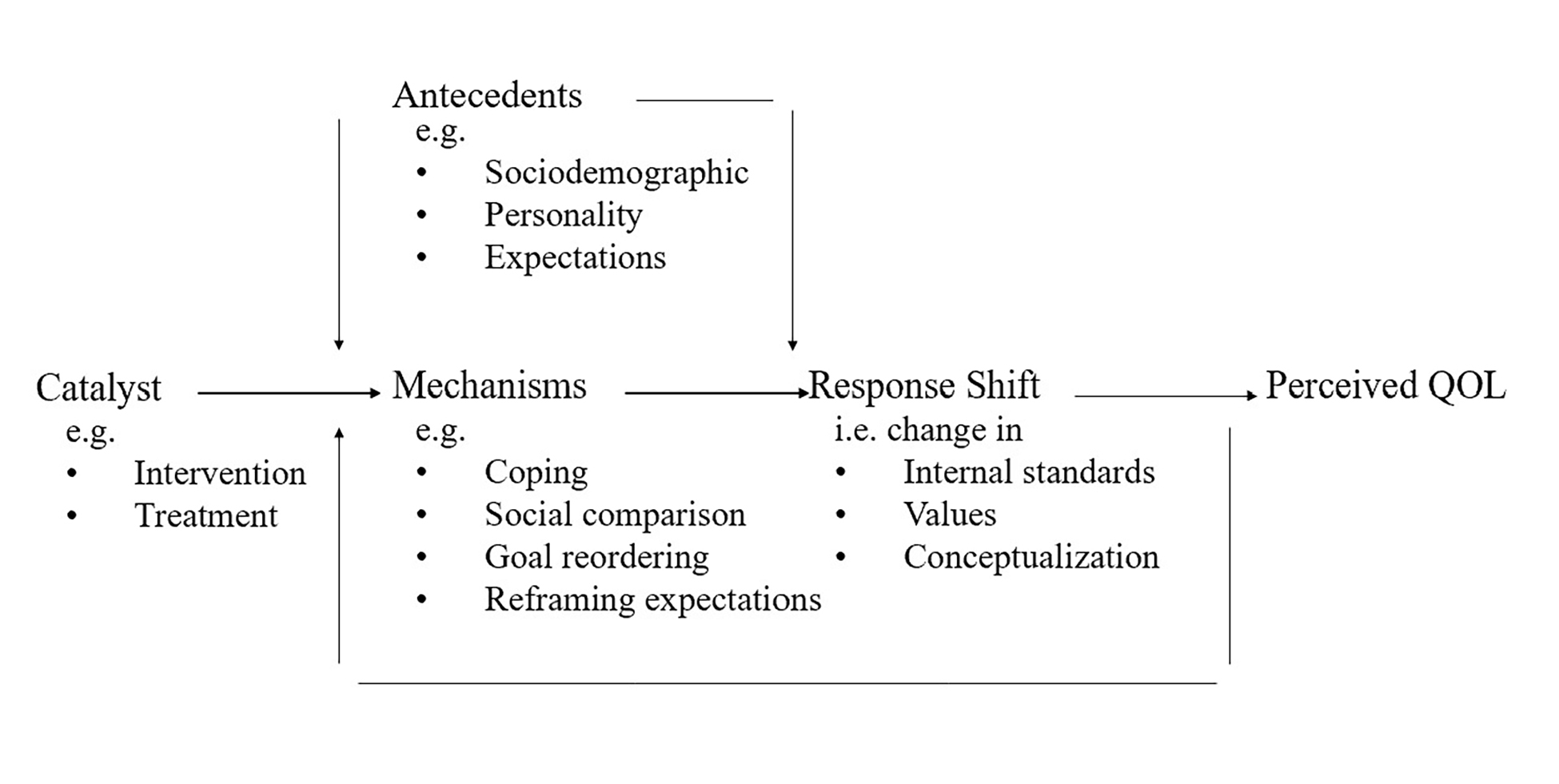

Supplement: Supplementary file 1 — Additional file 1: Figure S1. Theoretical model of RS. [file 12955_2021_1732_MOESM1_ESM.jpg]
