# Supplementary material for: Detecting response shift in health-related quality of life measurement among patients with hypertension using structural equation modeling
Source: Health Qual Life Outcomes. 2021 Mar 17;19:88. doi: 10.1186/s12955-021-01732-w (PMC7968327; doi:10.1186/s12955-021-01732-w)
Supplement: Supplementary file 2 — Additional file 2: Table S1. The result of selection bias test at baseline. [file 12955_2021_1732_MOESM2_ESM.docx]

Supplemental Table S1. Comparison of demographic characteristics, health conditions, and quality of life of the attrition sample with those of the analytic sample.

| **Variables** | | **Attrition sample** (N=37) | **Analytic sample** (N=203) | | **Test**  **statistic ^a^** | **P-value** |
| --- | --- | --- | --- | --- | --- | --- |
| ***Categorical variables* n (%)** | | | | | | |
| **Gender** | | | | | | |
| Male | | 16(43.2) | 109(53.7) | | χ^2^=2.509 | 0.113 |
| Female | | 21(56.8) | 94(46.3) | |  |  |
| **Marital status** | | | | | | |
| Married /co-habiting | | 32（86.5） | 181（89.2） | | Fisher’s Exact | 0.117 |
| Other | | 5（13.5） | 22（10.8） | |  |  |
| **Education attainment** | | | | | | |
| No School | | 1(2.7) | 6(3.0) | | F=0.544 | 0.704 |
| Elementary School | | 12(32.4) | 55(27.4) | |  |  |
| Middle School | | 13(35.1) | 60(29.9) | |  |  |
| High School or vocational training | | 7(18.9) | 41(20.4) | |  |  |
| College or above | | 4(10.8) | 39(19.4) | |  |  |
| **Employment status** | | | | | | |
| Employed | | 2(5.4) | 30(15.7) | | F=3.505 | **0.032^b^** |
| Not employed/ Retired | | 30(81.1) | 153(80.1) | |  |  |
| Full-time housework | | 5(13.5) | 8(4.1) | |  |  |
| **Annual household income (Yuan，1 USD = 6.8 RMB)** | | | | | | |
| <60,000 RMB | | 30(81.1) | 170(83.7) | | χ^2^=0.911 | 0.34 |
| ≥60,000 RMB | | 7(18.9) | 33(16.3) | |  |  |
| **Experienced severe illness** | | | | | | |
| Positive | | 15(40.5) | 71(35.1) | | χ^2^=1.005 | 0.316 |
| Negative | | 22(59.5) | 131(64.9) | |  |  |
| **Family members experienced severe illness** | | | | | | |
| Positive | 14(37.8) | | | 68(33.8) | χ^2^=0.559 | 0.455 |
| Negative | 23(62.2) | | | 133(66.2) |  |  |
| **Duration of hypertension** | | | | | | |
| < 6 months | | 2(5.4) | 16(7.9) | | Fisher’s Exact | 0.745 |
| ≥6 months | | 35(94.6) | 187 (92.1) | |  |  |
| **Last month blood pressure** | | | | | | |
| <140/90mmHg | | 14(48.3) | 110(55.3) | | χ^2^=0.500 | 0.479 |
| ≥140/90mmHg | | 15(51.7) | 89(44.7) | |  |  |
| **Health risk level** | | | | | | |
| Low | | 9(34.6) | 51(27.4) | | χ^2^=0.377 | 0.769 |
| Moderate | | 14 (53.8) | 100(53.8) | |  |  |
| High | | 2(7.7) | 26 (14.0) | |  |  |
| Very high | | 1(3.8) | 9(4.8) | |  |  |
| **Anti-hypertensive medications** | | | | | | |
| 0 | | 1(3.2) | 16(7.9) | | χ^2^=2.598 | 0.053 |
| 1 | | 27(87.1) | 134(66.3) | |  |  |
| 2 | | 1(3.2) | 44(21.8) | |  |  |
| ≥3 | | 2(6.5) | 8(4.0) | |  |  |
| ***Continuous variables* Mean (SD)** | | | | | | |
| **Age** | | 69.3(16.8) | | 65.9(10.8) | t=-1.231 | 0.225 |
| **PF** | | 60.8(33.5) | | 74.7(24.1) | t=2.283 | **0.028** |
| **RP** | | 45.8(47.6) | | 72.7(41.1) | t=2.647 | **0.013** |
| **BP** | | 74.4(21.1) | | 77.8(21.5) | t=0.851 | 0.396 |
| **GH** | | 50.3(23.5) | | 54.4(19.4) | t=1.089 | 0.277 |
| **VT** | | 52.0(16.3) | | 55.1(18.8) | t=0.875 | 0.383 |
| **SF** | | 78.8(22.9) | | 80.0(20.5) | t=0.322 | 0.748 |
| **RE** | | 73.3(44.1) | | 84.1(33.9) | t=1.060 | 0.301 |
| **MH** | | 68.9(20.6) | | 70.0(20.9) | t=0.267 | 0.790 |

^a^ One-way ANOVA, chi-squared or Fisher’s exact tests were used for categorical data, and t-tests were used for continuous data.

^b^ Result of Post Hoc Multiple Comparisons shows the proportion of patients with fulltime housework was significantly different among the two samples.
